# Immunomodulation in non-alcoholic fatty liver disease: exploring mechanisms and applications

**DOI:** 10.3389/fimmu.2024.1336493

**Published:** 2024-01-30

**Authors:** Ziwei Guo, Qinjuan Wu, Pengfei Xie, Jiuchong Wang, Wenliang Lv

**Affiliations:** ^1^ Department of Infection, Guang’anmen Hospital, China Academy of Chinese Medical Sciences, Beijing, China; ^2^ Guang'anmen Hospital, Beijing University of Chinese Medicine, Beijing, China

**Keywords:** immunoregulation, non-alcoholic fatty liver disease, NASH, immune cells, Kupffer cells

## Abstract

Non-alcoholic fatty liver disease (NAFLD) exhibits increased lipid enrichment in hepatocytes. The spectrum of this disease includes stages such as nonalcoholic simple fatty liver (NAFL), nonalcoholic steatohepatitis (NASH), and liver fibrosis. Changes in lifestyle behaviors have been a major factor contributing to the increased cases of NAFLD patients globally. Therefore, it is imperative to explore the pathogenesis of NAFLD, identify therapeutic targets, and develop new strategies to improve the clinical management of the disease. Immunoregulation is a strategy through which the organism recognizes and eliminates antigenic foreign bodies to maintain physiological homeostasis. In this process, multiple factors, including immune cells, signaling molecules, and cytokines, play a role in governing the evolution of NAFLD. This review seeks to encapsulate the advancements in research regarding immune regulation in NAFLD, spanning from underlying mechanisms to practical applications.

## 1 Introduction

**Figure 1:**
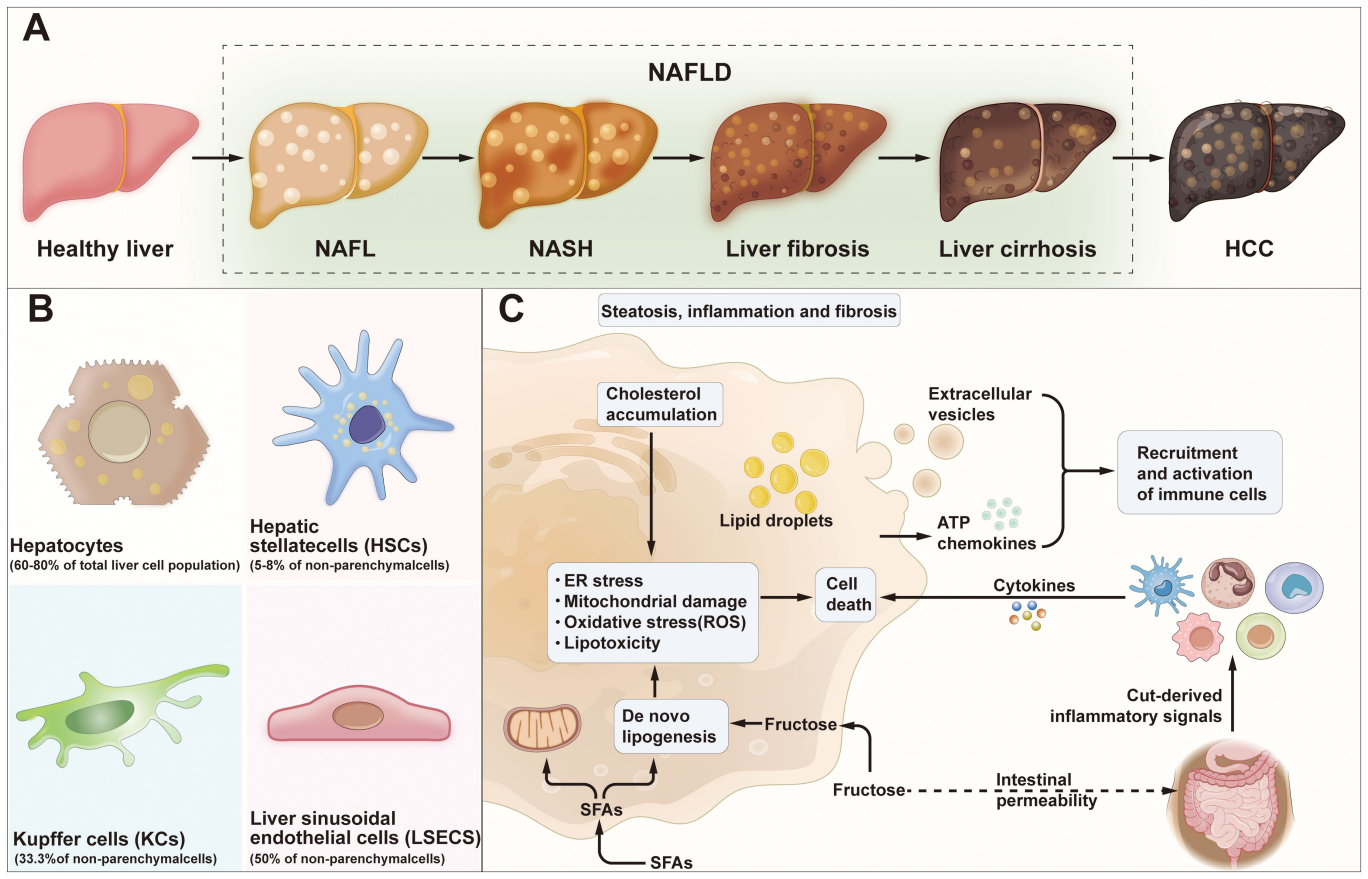
NAFLD progression and cell-to-cell signaling. **(A)** Development and progression of Healthy liver to HCC. Healthy liver can develop into NAFL, NASH, liver fibrosis, liver cirrhosis and/or HCC due to several factors. **(B)** HCC: hepatocellular carcinoma; Major cell type distribution in the liver and their known functions. **(C)** Pathogenesis of NAFLD. NAFLD, non-alcoholic fatty liver disease; NAFL, non-alcoholic fatty liver; NASH, non-alcoholic steatohepatitis; SFAs, saturated fatty acids; ER stress, endoplasmic reticulum stress; ATP, adenosine-triphosphate.

Non-alcoholic fatty liver disease (NAFLD) is a condition that manifests as excessive fat enrichment in the liver parenchyma cells, in individuals not taking alcohol (1, 2). NAFLD is the most frequent liver disease globally (3, 4), with a global prevalence estimated to be 30.1%, a prevalence of 28.02% in Asia Pacific (5). It has been reported that NAFLD may progress to several conditions including HCC, liver fibrosis, and NASH (6–9). Over the last three years, the terminology for NAFLD has undergone two revisions. Initially, an international panel of experts, in a publication in Gastroenterology in May 2020, proposed changing its name to metabolic dysfunction-associated fatty liver disease (MAFLD) (10–12), and then in June 2023, the European Society of Hepatology published the ‘Multi-Society Delphi Consensus on the New Nomenclature of Steatohepatitic Liver Disease’, which proposed the renaming of NAFLD to metabolic dysfunction-associated steatotic liver disease (MASLD) (10). Currently, there is no consensus concerning the naming of NAFLD, MAFLD, and MASLD. Therefore, these three naming systems are concurrently used. The occurrence of NAFLD is driven by several metabolic, genetic, environmental, and gut microbiological factors, and its pathogenesis has shifted from the traditional ‘double whammy’ model to a ‘multiple damage’ model (11–13). In terms of mechanisms, NAFLD may be caused by lipotoxicity arising from impaired lipid metabolism, recruitment and activation of inflammatory cells due to oxidative stress and increased endoplasmic reticulum stress (14–17). In addition, dysbiosis of the intestinal flora and the ‘gut-liver axis’ have been ascribed to the exacerbation of hepatic inflammation (18–20). Currently, there are no effective pharmacological treatments for NAFLD. Therefore, therapeutic strategies primarily focus on lifestyle modifications, weight loss, and management of muscle wasting syndromes to improve insulin resistance and control NAFLD progression (21). Investigating the mechanisms leading to NAFLD is crucial to the design of new drug targets as well as non-invasive tests (NIT) for steatohepatitis and fibrosis. It may also reveal important markers for monitoring the prognosis of the disease (11)(**Figure 1**).

Immunomodulation is a process in which the body maintains immune homeostasis and restores normal immune response in response to external stimuli. Several factors are involved in this process including immune cells, signaling molecules and cytokines (22). The immune system constitutes the innate (general, nonspecific) immune system and the adaptive (specialized) immune system (23, 24). Intrinsic immunity is the natural immune defense function established by the organism during development and evolution, and forms the basis of all immune responses, also known as non-specific immunity (25, 26). The innate immune system’s primary defense mechanisms include natural killer cells, macrophages, and neutrophils. In contrast, adaptive immunity enables the organism to identify and eliminate foreign substances. This process involves T-cell-mediated cellular immune and B-cell-mediated humoral responses. Both responses rely on the T cell receptor (TCR) and B cell receptor (BCR) to recognize a vast array of antigenic molecules. Enhancing adaptive immunity can effectively boost autoimmune function (27). Recently, significant breakthroughs have been reported in the field of immunomodulation. For instance, many new regulatory mechanisms have been discovered in T cells (28, 29), B cells (30), and dendritic cells (DCs) (31, 32). The liver, acknowledged as a crucial immune organ, harbors a diverse spectrum of both innate and adaptive immune cells (33, 34). These immune cells play a pivotal role in modulating the inflammatory response throughout the development and progression of NAFLD by releasing essential cytokines, such as monocyte chemotactic protein and tumor necrosis factor (35, 36). Increased levels of fatty acids in the local hepatic microenvironment reduces the number of CD4^+^ T lymphocytes. Hence, the absence of CD4^+^ T lymphocytes in the hepatic microenvironment may be a key factor leading to impaired immune regulation during NAFLD progression to cirrhosis and liver cancer (37). Immune cells influence liver fibrosis initiation by modulating the activation of hepatic stellate cells (HSCs) (33).

Hence, immune cells, signaling molecules, and cytokines within the immune system participate in the progression of NAFLD, collectively exerting significant immunomodulatory effects. The primary international research consortia in the field of NAFLD, such as NIMBLE and LITMUS, have primarily focused on screening and validating metabolic biomarkers, with a comparative lack in investigating the immunological mechanisms during disease progression (34, 38, 39).However, an increasing number of basic research studies are recently turning their attention to this area. One study compared the phenotypic changes in BALB/c mice resistant to NASH and Rag gene knockout mice under the NASH model. The study found increased activation of liver T cells in BALB/c mice, while the NASH condition worsened in Rag gene knockout mice, suggesting the role of adaptive immunity in NASH development (40).Therefore, a thorough analysis of the precise roles of different immune cell populations in the pathogenesis of NAFLD, and using this as a critical first step for therapeutic intervention, represents a crucial starting point for immunotherapy. This review seeks to present a comprehensive summary of recent advancements in comprehending immune regulation in NAFLD. Furthermore, we will encapsulate ongoing research efforts that concentrate on therapeutic strategies aiming to address NAFLD through immune modulation.

## 2 Innate immune cells and NAFLD

### 2.1 Macrophages and NAFLD

Macrophages are formed from yolk sac progenitor cells and possess self-renewal characteristics. Hepatic macrophages act as storage macrophages in the liver, accounting for more than 80% of systemic tissue macrophages, the most representative of which are Kupffer cells (KCs) (41). These cells, constituting 15% of the liver’s total cell population, are highly mobile hepatic macrophages situated on the endothelial side of the blood sinusoids (42, 43). Kupffer cells (KCs) are differentiated from monocyte-derived macrophages (MoDMacs) by their distinctive localization and swift accumulation in the injured liver (43). Similar to resident tissue macrophages, KCs possess a mature phenotype and exhibit significant plasticity. Their functional activity changes in response to changes in metabolism and local immune state (42). Macrophages are divided into two distinct phenotypes: M_1_ and M_2_. M_1_ macrophages, which are referred to as activated macrophages that can potentially release massive amounts of pro-inflammatory cytokines. Conversely, M2 macrophages, often referred to as activated macrophages, exhibit a tendency to release diminished quantities of pro-inflammatory cytokines while increasing levels of anti-inflammatory mediators (43, 44).

Hepatic macrophages are pivotal in preserving homeostasis through interactions with hepatocytes, HSCs, and hepatic sinusoidal endothelial cells. While they participate in maintaining tolerance and recognizing and eliminating exogenous material such as circulating cellular debris, pathogens, or apoptotic cells, they also contribute significantly to disease progression. They are involved in erythrocyte clearance and systemic iron and cholesterol metabolism, and can trigger cell apoptosis in some cells (45). However, as immune cells, they can regulate the recruitment of other immune cells, with macrophages recognizing pathogen-associated molecular patterns (PAMPs) or damage-associated molecular patterns (DAMPs) via pattern recognition receptors (PRRs) on the cell surface. Thus, they secrete the production of pro-inflammatory and anti-inflammatory cytokines, chemokines, and other molecules, recruit and activate intrinsic immune cells and an adaptive immune responses (45, 46). In the liver, macrophage transcription profiles are characterized by an enrichment of genes associated with lipid metabolism functions, including CD36 (47) and Liver X Receptor Alpha (LXRα) (48). Hepatic macrophages actively contribute to the pathogenesis of nonalcoholic steatohepatitis (NASH) (49–52). Several diet-induced animal models of NASH have demonstrated that depleting macrophages, using methods such as clodronate liposomes or gadolinium chloride, effectively mitigates hepatocyte steatosis, necrotic inflammation, and interstitial fibrosis (53). However, application of sc-RNA seq has uncovered high heterogeneity in hepatic macrophages, suggesting that liver-resident hepatic macrophages and MoDMacs play diverse roles in the regulation of hepatic inflammation and NAFLD progression to NASH (47, 54).In a study concerning metabolic-associated fatty liver disease (MAFLD), a reduction in KCs was observed among MAFLD patients, which were replaced by newly emerged macrophages originating from the bone marrow. These newly emerged macrophages exhibit two distinct subgroups: one resembling KCs under healthy conditions, and the other sharing similarities with LAMs found in obese adipose tissue. This study unveils the diversity of intrahepatic macrophages (48).In the context of NASH, KCs derived from monocytes exhibit functional differences compared to the original KCs and have distinct impacts on NASH pathology (49).

**Figure 2:**
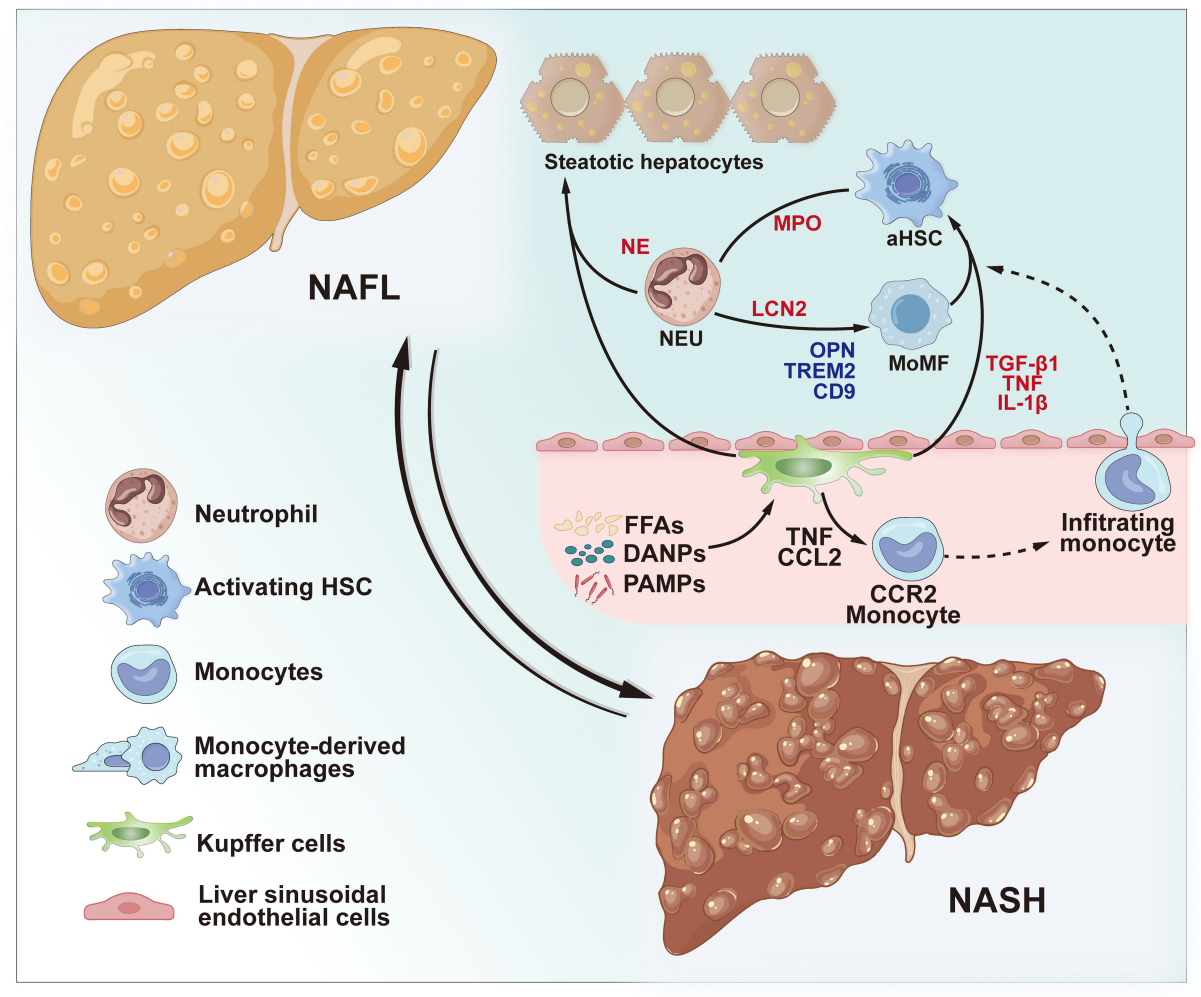
Role of innate immune cells in NAFLD and NASH. During the progression of non-alcoholic steatohepatitis NASH, KCs can be activated by circulating FFAs and cytokines from inflamed obese adipose tissue, PAMPs from the microbiota, and DAMPs released by damaged hepatocytes. Activated KCs contribute to hepatic steatosis by influencing lipid metabolism in hepatocytes. Furthermore, activated KCs amplify the inflammatory response by secreting TNF and CCL2, thereby enhancing the CCR2-dependent recruitment of monocytes into the liver. Infiltrating monocytes, in turn, secrete pro-inflammatory MoMFs, fostering the progression of NASH and fibrosis. The cytokines TNF, IL-1β, and TGF-β1 produced by activated KCs and macrophages contribute to the activation of HSCs. Neutrophils are recruited to the liver and accumulate in chronically inflamed tissues during NASH. NETosis and neutrophil activation lead to the release of granule proteins, including NE, LCN2, and MPO thereby contributing to hepatic steatosis, increased inflammation, and fibrosis. NAFL, non-alcoholic fatty liver; NASH, non-alcoholic steatohepatitis; KCs, Kupffer cells; PAMPs, pathogen-associated molecular patterns; DAMPs, damage-associated molecular patterns; TNF, tumor necrosis factor; CCL2, chemokine (C-C motif) ligand 2; CCR2, C-C chemokine receptor type 2; MoMFs, monocyte-derived macrophages; IL-1β, interleukin 1 beta; TGF-β1, transforming growth factor-beta; HSCs, hepatic stellate cells; NE, neutrophil elastase; MPO, myeloperoxidase; LCN2, lipocalin 2.

In the early stages of NAFLD, free fatty acids, trans fatty acids, and peroxidized fatty acids from fat activate macrophages via the Toll receptor signaling pathway (50–52). The imbalance in gut microflora and impaired gut barrier function result in the generation of bacterial products, including endotoxin. These products circulate to the liver through the portal vein, activating macrophages. This activation leads to an increased pro-inflammatory polarization of macrophages, fostering hepatic steatosis, initiating inflammation, and facilitating the recruitment of other immune cells into the liver (55). Moreover, the secretion of IL-1b by pro-inflammatory macrophages inhibits peroxisome proliferator-activated receptor α-mediated fat oxidation, leading to the accumulation of triglyceride in hepatocytes (56). Therefore, in NASH, the combination of lipotoxicity and the release of inflammatory cytokines trigger hepatocyte necrosis. Subsequently, these necrotic hepatocytes produce DAMPs, which further stimulate macrophages. This sets off a positive feedback loop, exacerbating hepatic injury (57). Depletion of macrophages ameliorated hepatic insulin resistance and hepatic steatosis in rats fed a high-sugar or high-fat diet (58). Upon activation, macrophages undergo differentiation into NASH-associated KCs, secreting chemokines like CCL2. This secretion results in the recruitment of MoDMacs to the damaged liver. In the context of NASH, activated KCs/macrophages produce pro-inflammatory cytokines. These cytokines, in turn, contribute to the survival of activated HSCs via the NF-κB signaling pathway. Moreover, activated macrophages have been reported to enhance the differentiation of HSCs into pro-fibrotic myofibroblastic cells by producing the transforming growth factor β1(TGF-β1), promoting collagen deposition, leading to hepatic fibrosis (59) (**Figure 2**).Adipose tissue macrophages constitute a critical cell population influencing the development of NAFLD, which is with obesity being a significant risk factor for non-alcoholic fatty liver disease. In mice fed a high-fat, high-sugar diet, genes for macrophage recruitment and inflammation (such as Ccl2, Ccr2, Il1b, and Tnfa) exhibit earlier upregulation in adipose tissue compared to the liver. In complex mouse models of obesity and NASH, pro-inflammatory macrophages in abdominal adipose tissue produce substantial amounts of neutrophil chemotactic proteins. This leads to increased infiltration of neutrophils and macrophages in the liver, exacerbating liver injury. Additionally, studies have highlighted a relationship between the quantity of adipose tissue macrophages and the severity of liver inflammation and fibrosis (60, 61).

To date, the phenotypes of macrophages are not standardized, and there is no consensus concerning whether the macrophages in NASH arise from a phenotypic shift in resident KCs or from newly recruited macrophages. Additional markers and distinct macrophage populations are expected to be identified in the future. The phenotypic alterations and functional roles of newly recruited macrophages in the NASH microenvironment remain unclear. Studies have indicated that, with the progression of NAFLD, the resident KCs diminish, being substituted by MoMFs and NASH-associated KCs—a more pro-inflammatory pool that exacerbates liver injury during NASH. Studies have shown that macrophages exhibit anti-inflammatory phenotype and may activate of M1 macrophages. This mechanism can alleviate NAFLD-associated liver injury in mice (54). Utilizing single-cell profiling and spatial proteomics techniques has unveiled the distinct spatial distribution of hepatic macrophages and adipose-related macrophages within the liver, shedding light on the microenvironmental circuits steering their unique transcriptional characteristics. For example, lipid-associated macrophages (LAMs) within the bile duct region are induced by local lipid exposure, while the development of Kupffer cells critically depends on the interaction between the ALK1-BMP9/10 axis and hepatic stellate cells (62).Hepatic macrophages, originating from various sources, display significant heterogeneity and evolve with the progression of NAFLD, revealing limitations and shortcomings in prior research. Further investigations are needed to elucidate the phenotype, origin, localization, and function of liver macrophages. We postulate that the subtypes, cell-cell interactions, cellular signal transduction, epigenetic features, cellular metabolic status, and functions of hepatic macrophages can be more comprehensively clarified through the integration of single-cell RNA sequencing with advanced technologies such as multi-omics (63, 64).

### 2.2 Neutrophils and dendritic cells

Besides KCs, other innate immune cells such as, neutrophils, dendritic cells (DCs), and lymphocytes in the liver can collaborate to create a highly coordinated network. This network can detect pathogens, PAMPs or DAMPs, and increase metabolite levels, hence activate inflammatory reactions and metabolic disruptions which promote NAFLD progression. Neutrophils are considered to be major factors that activate NASH development (56, 57). Metabolic injury in the liver, coupled with bacterial overgrowth, intensifies the activation and mobilization of neutrophils. This process triggers the activation of KCs and endothelial cells, prompting the upregulation of cell adhesion molecules and facilitating the recruitment of other downstream cells. In a rodent model, depletion of neutrophils attenuated hepatic lipid accumulation and inflammation induced by a HFD, thereby hindering the progression of NASH (65). Activated neutrophils secrete myeloperoxidase (MPO), neutrophil elastase (NE), protease 3, histatin and matrix metalloproteinase 9 (MMP-9), all of which increase oxidative stress and promote liver injury (66–69). Among them, MPO is mainly produced by the polymorphonuclear neutrophils (NEU) which play important roles in lipid peroxidation in various inflammatory diseases (70). MPO facilitates the establishment of mitochondrial permeability transition pores, which lead to the death of hepatocyte (71). Oxidative stress initiated by MPO could increase DNA damage and formation of genomic mutations, hence elevating malignant development in NAFLD (72). Elastase, on the other hand, modulates hepatic insulin resistance by enhancing the establishment of inflammatory responses. Therefore, research has demonstrated that deficiency of neutrophil elastase ameliorates hepatic inflammation (63). Neutrophils can also activate TLR2 and TLR4 receptors, hydrolyze lysosomes and proteins in the extracellular matrix (ECM). Activation of TLR4 signaling in hepatic macrophages have been reported to promote neutrophil adhesion to hepatic sinusoids, and neutrophils also enhance macrophage recruitment via antigen presentation. In addition, neutrophils increases the occurrence of fibrosis by activating and promoting HSCs proliferation via MPO and MMP-9 (64).

Neutrophil extracellular traps (NETs) represent an immune response mechanism associated with neutrophils. These fibrous networks, primarily composed of DNA and adorned with antimicrobial peptides and enzymes, are released into the extracellular space by neutrophils. They effectively capture and eradicate pathogens such as bacteria and viruses, constituting a critical part of the body’s innate immune system. Despite their pivotal role in the body’s immune defense, excessive activation of NETs can lead to inflammation and damage in surrounding tissues. In the progression of NASH, excessive lipid accumulation in liver tissues triggers an inflammatory response, prompting host cells to release neutrophil chemotactic factors like IL-8. This stimulates neutrophil migration to the liver and their activation, potentially causing excessive NETs activation and release. Consequently, this induces immune cell infiltration and activates inflammatory pathways in the liver, exacerbating NASH (73, 74).Studies by Dirk J. et al. observed a significant increase in serum NETs levels in NASH patients and validated in NASH animal models NETs could promote the progression of HCC (65).Additionally, Wang et al. revealed in non-alcoholic steatohepatitis (NASH) that NETs promote Tregs activity through metabolic reprogramming, inhibiting early immune surveillance of NASH and driving the development of HCC (75).

DCs possess the ability to sense local conditions, recognize pathogens, and respond to danger signals. The phenotype of hepatic DCs can transition from a tolerant state to an adaptive state based on the liver microenvironment and intracellular lipid content. Hepatic dendritic cells (HDCs) play a role in the regulation of lymphocyte antigen presentation and hepatic immune responses. Studies have reported that HDCs have pro-inflammatory roles, and depletion of CD11c^+^DC or CD103^+^DC inhibits the level of inflammation-promoting cytokines and chemokine expression, which reduces liver fat content and chemokines levels, eventually decreasing the reduction of liver fat content (76). However, other studies have demonstrated that CD11c^+^DC exert protective effects against MCD-induced fibro inflammation and regulated the neutral function in models of choledochal ligation and carbon tetrachloride-induced hepatic fibrosis. The inconsistent findings could stem from variations in NASH animal models, as previous studies have suggested that dietary factors can impact the phenotypic switching of distinct DC populations. However, the specific role of DCs in NASH remains unclear (77). A recent investigation which employed single-cell transcriptomics indicated a marked elevation in hepatic conventional dendritic cells (cDCs), which are important pathological markers. Furthermore, they found that these cDCs originated from an augmented circulation of bone marrow cDC progenitor cells induced by NASH. Examination of blood and liver samples from individuals across the spectrum of NAFLD/NASH revealed elevated levels and heightened activity of type 1 conventional dendritic cells (cDC1) within the disease milieu. Sequencing analysis of physically interacting cDC-T cell pairs from liver-draining lymph nodes suggested that cDCs in NASH played a role in exacerbating the condition by promoting inflammatory T cell reprogramming. Ultimately, in a mouse model of NASH (XCR1DTA mice), knockdown of cDC1 resulted in the attenuation of liver pathology (78).

## 3 Adaptive immunity and NAFLD

Evidence from numerous studies has shown that B-lymphocyte and T-lymphocyte modulate adaptive immunity of NAFLD and NASH (58, 71, 79). These stem cells, referred to as pluripotent stem cells, possess the ability to self-replicate and differentiate as required by the body, undergoing division and proliferation when needed. When influenced by hormonal stimulation, a subset of these cells undergoes differentiation into lymphoid stem cells, which then give rise to T cells and B cells. Studies have indicated that 60% of NASH patients exhibit localized accumulations of both B and T cells in the liver. It should be noted that the size and prevalence of these aggregates are positively correlated with lobular inflammation and fibrosis scores (80).

### 3.1 B-lymphocytes

B cells generate proinflammatory mediators and act as antigen presenters that promote T-cell and macrophage activation as well as regulate the initiation of inflammatory response and hepatic fibrosis in NASH. Research has shown that B1 and B2 cells might have contrasting roles in the development of NAFLD, with B1 cells functioning as anti-inflammatory agents through the production of IgM targeting oxidized low-density lipoprotein (ox-LDL), while B2 cells contribute to proinflammatory effects by releasing IgG. B2 cells have multiple roles such as, anti-inflammatory function, possibly arising from this very trait. B1 cells produce IgM, which targets oxidized low-density lipoprotein (ox-LDL) to exert anti-inflammatory effects. Conversely, B2 cells, responsible for IgG production, tend to exert pro-inflammatory effects. In a mouse model of NASH, CD43^-^CD23^+^B2 cells were reported to regulate the adaptive immune response, whereas the expression of B-cell activating factor was upregulated in the liver. The concentration of CD19+CD24hiCD38hi B cells in the circulating blood is associated with the extent of steatosis and fibrosis (81). In NASH patients, the activation of B cells leads to elevated expression of major histocompatibility complex II, and their migration to the liver precedes the recruitment of CD4+ and CD8+ T cells. Inhibition of B2 cells suppresses hepatic Th1 activation and IFN-γ expression (72). B cells enhance the expression of pro-inflammatory agents like IL-8, IL-6, and TNF-α, while decreasing the expression of IL-10. This triggers pro-inflammatory and pro-fibrotic responses in HSCs and macrophages. At the same time, activated HSCs support the survival and development of hepatic B cells by releasing retinoic acid (82). B cell antigen presentation can insulin resistance, ameliorate hepatic steatoinflammation, and insulin resistance in a mouse model of B2 cell deficiency or dysfunction. In another study, transplantation of gut microbiota from patients with NAFLD into recipient mice was found to accelerate the advancement of NASH. This acceleration was attributed to the heightened accumulation and activation of intrahepatic B cells, indicating that gut microbial factors play a role in the pathogenic activity of B cells during the advancement of NASH (83).

Recent studies have highlighted the crucial role of dysfunctional intestinal B cells in the pathogenesis of NASH. It has been observed that intestinal B cells in NASH mice can directly activate CD8+ T cells, instigating an immune response. This B cell-mediated activation of CD8+ T cells is antigen-independent and does not rely on the gut microbiota. In experiments where researchers depleted B cells in the NASH mouse model using B cell-specific antibodies or genetic engineering methods, they observed a significant decrease in inflammation and fibrotic markers in the liver tissues of mice. This implies that depleting B cells could effectively prevent or reverse NASH and liver fibrosis. Concurrently, researchers identified elevated levels of IgA in the serum of NASH patients and demonstrated that IgA secretion contributes to liver fibrosis by activating the FcRc signaling pathway in specific immune cells (84).

Although B lymphocytes participate in inflammatory conditions, given their capacity to produce antibodies and cytokines, their participation in NAFLD and NASH remains relatively unexplored. In future, both antigen-dependent and independent pathways that activate intrahepatic B cells and their contribution to NASH, particularly in the activation of HSCs, should be investigated. Advances in monocytomics can reveal the functional significance of the B1 subset in disease progression (85).

### 3.2 T-lymphocytes

T-lymphocytes consist of several subpopulations. They can be divided into various subpopulations based on their surface markers and functional characteristics. Moreover, each subpopulation regulates and synergizes the other in regulating the immune function. Based on the expression level of CD molecules, they are classified into CD4^+^ T lymphocytes and CD8^+^ T lymphocytes. Regarding cellular functional characteristics, they can be classified into helper T cells (Th), regulatory T lymphocytes (Tregs) and cytotoxic T lymphocytes (CTL), of which Th cells can be differentiated into different functional subpopulations, such as Th0, Th1, Th2, Th17 and Tfh (77, 86).

M1 macrophages enhance the differentiation of CD4^+^ T cells into T helper 1 (Th1) cells. Researchers have shown that liver Th1 cell levels are increased in patients with NASH, and IFN-γ levels are positively correlated with the size and population of hepatic lymphocyte aggregates and fibrosis severity (72). Th1 cells, recognized for their pro-inflammatory characteristics, promote hepatic inflammation by elevating the production of cytokines such as IL-12 and TNF-α. Studies indicate increased levels of Th1 cells in the peripheral blood of NAFLD patients. *In vitro* experiments suggest that exposure to hepatic fat toxicity induces the differentiation of naive T cells towards Th1 cells. Th1 cells primarily induce tissue damage by secreting inflammatory cytokines like IFN-γ. Additionally, IFN-γ has the capability to activate macrophages, amplifying their pathogenic role (87).In the later phases of NASH in a mouse model with a choline-methionine-deficient diet (MCD), increased hepatic Th1 cell levels contribute to the advancement of fibrosis. Animal models of NAFLD showed enhanced Th17 phenotype in the liver, which promoted inflammation (36). This is consistent with findings from a recent study, which proposed that the systemic presence of IL-17, induced by Segmented Filamentous Bacteria (SFB), enhances systemic inflammation, leads to adverse physiological outcomes, and worsens NAFLD in obese individuals. In a NAFLD model affected by both diet-induced intestinal damage and ecological imbalances, a more inflammatory Th17 cell phenotype may be favored. It is further hypothesized that the colonization by pathogenic flora induces a high degree of adaptability, pro-inflammatory cytokine production, and a metabolic inclination towards aerobic glycolysis and oxidative phosphorylation in Th17 cells (88). Th17 and IL-17 have been linked to the occurrence of IR and steatohepatitis. and steatohepatitis.

**Figure 3:**
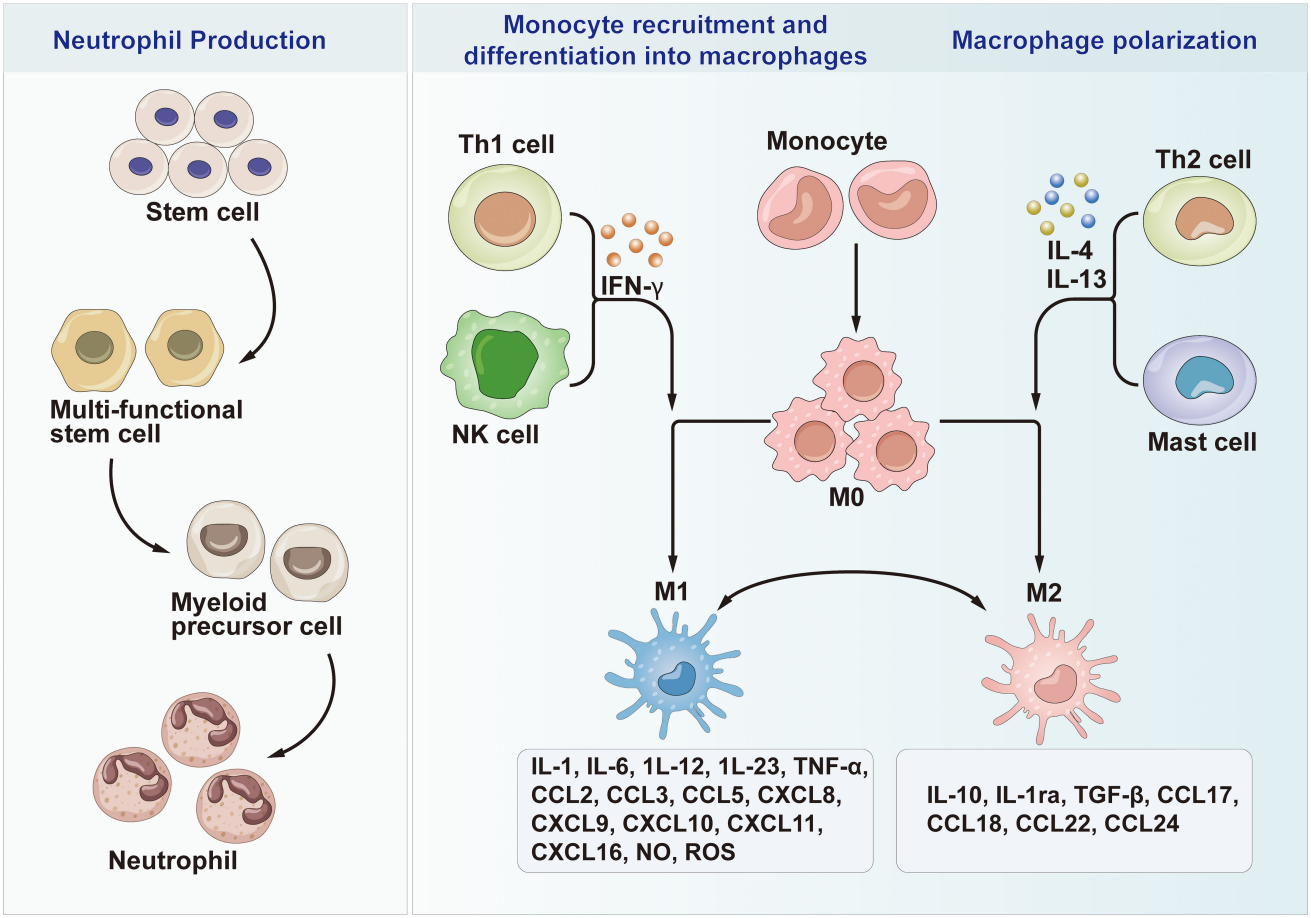
Neutrophil Production, Monocyte recruitment and differentiation into macrophages and Macrophage polarization. Th1, T helper cell 1; Th2, T helper cell 2; M0, Macrophages 0; M1, Macrophages 1; M2, Macrophages 2; IL, Interleukin; CCL, chemotactic cytokines; CXCL, C-X-C motif chemokine ligand; NK cells, Natural killer T cells; IFN-γ, Interferon-γ; TGF-β, Transforming growth factor-β; TNF-α, Tumor necrosis factor-alpha; NO, Nitric oxide; ROS, Reactive oxygen species.

Regulatory T cells (Tregs) are a subset of T cells which inhibit the forkhead-like transcription factor (FOXP3). In the liver, DCs drive the differentiation of CD4^+^ T cells to Treg by expressing programmed cell death ligand 1 (PD-L1) and secreting IL-10. While their exact contribution to the initiation and progression of NAFLD is not fully elucidated, the significance of intrahepatic Tregs in NASH remains unclear. However, it is established that they play a role in maintaining immune response balance by inhibiting the proliferation and activation of cytotoxic T cells. Tregs have can prevent autoimmune hepatitis by proliferation of self-reactive cells, as well as in the NASH immune response and negative control of organ tolerance during transplantation (80, 81, 89). Treg cells are involved in the regulation of immune tolerance in the liver by directly inhibiting the proliferation and function of CD4+ and CD8+ T cells. In patients with NAFLD, the levels of Treg cells are lower compared to those in healthy subjects. In pathological conditions like NASH, Tregs exhibit increased vulnerability to apoptosis, potentially influenced by local ROS, the immunosuppressive role of DCs, or the IL-33-mediated disturbance in the differentiation signals of Tregs. Tregs inhibit fibrosis by secreting IL-10, and hence Tregs may inhibit the development of NASH (85, 90). In addition, a previous study indicated that induction of Tregs reduced insulin resistance and thus ameliorated liver injury. A study investigating the effects of Tregs using the immunosuppressant OKT3 in patients with NASH reported that NASH patients exhibited improved immune parameters and liver function (91) (**Figure 3**).

Abundant infiltration of CD8+ T lymphocytes can be detected in the liver tissue of NAFLD patients. Recent single-cell RNA sequencing analysis of human liver tissue in NASH patients revealed a significant presence of hyperfunctional CXCR6+PD1high CD8+ T cells in the liver. These cells exhibit elevated expression of cytotoxicity-related genes and display autoaggressive characteristics, correlating positively with liver inflammation and fibrosis severity in patients. *In vitro* experiments suggest that these autoaggressive CD8+ T cells originate from IL-15-activated CD122+ T cells. In mouse models, selective depletion of CXCR6+CD8+ T cells in a mouse NASH model significantly improved liver pathological damage. a pivotal role of accumulated autoaggressive CD8+ T cells in the liver and their activating factor, IL-15, in the pathogenesis of NASH (92). CD8 T cells are closely associated with the development of liver fibrosis in NASH mouse models. Furthermore, a decrease in CD8 T cells in NASH liver is accompanied by reduced mRNA expression levels of fibrosis-related genes (including TGF-β, α-smooth muscle actin, collagen type 1α1, and collagen type 1α2). CD8 T cells expressing CXCR1 and CXCR6 activate hepatic stellate cells by secreting IL-10, CXCL4, and CXCL16, thus promoting the pathological progression of NASH (93).

## 4 Prospects for the application of immunomodulation in the treatment of NAFLD

Disease immunological factors contribute to NASH occurrence, and several studies have investigated strategies for leveraging the immune system to treat NAFLD (92). A recent study employed a phenotype-based high-throughput screen and techniques such as transcriptome sequencing and immunoprecipitation-mass spectrometry to uncover innate immunity factors driving NAFLD occurrence. They found that RING finger protein 13 (RNF13) controls lipid deposition in NAFLD by modulating the levels of two intrinsic immune system components: triple structural domain protein 29 (TRIM29) and stimulator of interferon genes protein (STING), ultimately contributing to the inflammatory responses. The research showcased an elevation in RNF13 levels in the livers of individuals with NASH. Additionally, depleting RNF13 in hepatocytes intensified diet-induced hepatic insulin resistance, steatosis, inflammation, cellular damage, and fibrosis in mice, with these effects being alleviated by RNF13 overexpression. Mechanistically, RNF13 has been considered to promote the proteasomal degradation of STING in a ubiquitination-dependent manner, suggesting it is a promising target for the treatment of NAFLD (94). Zhang et al. established several mice NASH models and superfused *in vitro*-induced transformed double-negative T cells at different time points. They found that exogenous double-negative T cells migrated to the liver and visceral adipose tissue, where inflammation was most pronounced in NASH mice. It also inhibited local Th17 cells. On the other hand, it selectively suppressed pro-inflammatory M1 macrophages without affecting inflammation-suppressing M2 macrophages, demonstrating that the double-negative T cells could selectively inhibit the specific immune cell subpopulations. This study not only reveals a novel immunoregulatory function of double-negative T cells, adding to our understanding of hepatic immunity, but also presents a promising cell therapy approach for diet-induced obesity, type 2 diabetes, and NASH. This holds great potential for future clinical applications (95). Moreover, recent single-cell transcriptomics studies in humans and mice have identified a subpopulation of hepatic macrophages marked by elevated TREM2 expression. This subpopulation is strongly linked to the advancement of various liver diseases, including NAFLD, liver cirrhosis, and HCC. TREM2 is a single transmembrane receptor of the immunoglobulin superfamily, initially detected in monocyte-derived dendritic cells and macrophages. To date, its exact function and regulatory mechanism in NAFLD and NASH lesions have not been clarified. The precise role of TREM2 in NAFLD and NASH lesions, as well as its underlying regulatory mechanisms, are not well understood. Recent reports have suggested that macrophages expressing TREM2 can preserve hepatic immune balance and prevent the progression of NASH. High levels of TREM2 expression stimulate macrophages can efficiently and promptly clear apoptotic hepatocytes—a phenomenon referred to as the ‘cellular burial effect.’ This unveils an important mechanism by which the body counteracts chronic hepatic inflammation and the development of NASH induced by obesity. Therefore, inhibiting TREM2 cleavage in macrophages to reinstate their capacity for clearing apoptotic hepatocytes could be a strategy for preventing and treating NAFLD (96).sTREM2, the soluble variant of TREM2, is generated through cleavage of the TREM2 protein under certain circumstances, releasing it into the extracellular space. Detection of sTREM2 levels offers an assessment of disease status and inflammation severity. Research by Tim et al. indicates that increased soluble TREM2 levels reflect heightened inflammation and disease progression in NASH. Compared to conventional laboratory markers, it shows superior performance in portraying liver pathological changes in NASH and serves as a new biomarker for evaluating the severity of the condition. Although studies have confirmed sTREM2’s potential value as a biomarker for NASH fibrosis, further investigation is needed to verify its reliability and accuracy (97).

According to Ping et al., 17β-HSD7 was markedly elevated in macrophages from mice with high-fat diet and methionine-choline-deficient diet-induced NAFLD, leading to the promotion of M1 polarization through the modulation of intracellular cholesterol content and activation of NLRP3 inflammatory vesicles. This in turn led to the development of NAFLD. Moreover, researchers performed additional screening of an FDA-approved drug library and identified fenretinide, a 17β-HSD7 inhibitor, which was experimentally demonstrated to mitigate hepatocyte lipid accumulation by enhancing macrophage M1 polarization. This study highlighted that 17β-HSD7 can modulate macrophage polarization and may be a promising target for NAFLD therapy (98). Another team constructed mannose-modified siRNA lipid nanoparticle targeting hepatic macrophages (mLNP-siHMGB1) which regulated hepatic macrophages to increase their anti-inflammatory properties by targeting hepatic macrophages through mannose receptors on the surface of macrophages and silencing the expression of the HMGB1 gene. It successfully mitigated inflammation in the NASH lobules, reduced macroglossal steatosis in the livers of mice, and restored liver function to normal levels in the NASH model mice, thus enhancing liver function. The study explored the therapeutic possibilities of combining lipid nanoparticles with the polyunsaturated fatty acid docosahexaenoic acid (DHA) in the treatment of NASH. These discoveries provide valuable insights and lay a solid scientific groundwork for the future development of therapeutic strategies for NASH (99).

Concurrent research indicates certain impediments to macrophage autophagy within the NAFLD microenvironment, which play a key regulatory role in the immunopathological progression of NAFLD. Wang et al. observed increased expression of macrophage HIF-1α and decreased autophagic function in NAFLD animal models and patient samples. In mouse models, further investigations revealed that HIF-1α activation inhibits macrophage autophagy, activates the NF-κB pathway, upregulates inflammatory factors, exacerbating macrophage inflammatory activation and liver injury. These findings elucidate the synergistic mechanism between HIF-1α and autophagy in macrophage activation and inflammation (100). In addition, research teams identified high expression of the phosphatidylserine receptor Tim-4 in NAFLD macrophages. Tim-4 activates AMPK-mediated autophagic response, degrading components of the NLRP3 inflammasome, thereby inhibiting inflammasome activation, IL-1β, IL-18 release, and alleviating liver injury in NAFLD. In summary, enhancing autophagic levels in hepatic macrophages may emerge as a novel therapeutic target for NAFLD treatment (101).

Cenicriviroc (CVC) is a CCR2/CCR5 antagonist. CCR2 and CCR5 act as receptors for pro-inflammatory chemokines, primarily involved in recruiting inflammatory cells, particularly monocytes. By antagonizing chemokine receptors, CVC mitigates the infiltration of inflammatory cells, thus reducing inflammation and injury in NAFLD/NASH livers. Additionally, CVC inhibits the activation of hepatic stellate cells and reduces the production of pro-fibrotic cytokines, thereby impeding the progression of NASH-related liver fibrosis (102).A study examining the anti-fibrotic effects of high-dose CVC treatment in a diet-induced mouse NASH model demonstrated that it inhibited the aggregation of Ly6Chigh bone marrow-derived macrophages in the liver. Despite the persistence of fatty liver, however, the 14-week high-dose CVC treatment substantially reduced the degree of liver fibrosis in mice. *In vitro* investigations revealed CVC’s direct inhibition of pro-fibrotic gene expression in mouse hepatic stellate cells (103).Consistently, existing clinical research further confirms the beneficial impact of CVC in combating liver fibrosis, maintaining improvement even with long-term usage, thereby suitable for patients in advanced stages of fibrosis (95).However, in the latest phase III clinical trial of CVC for treating liver fibrosis in adult NASH patients, CVC failed to demonstrate significant efficacy in treating liver fibrosis in NASH patients but still exhibited good safety and tolerability (96).

During NAFLD, an excess of saturated fatty acids can activate the TLR4 signaling pathway, causing the massive secretion of inflammatory factors such as tumor necrosis factor-α, interferon-γ, inducing lipid deposition in liver tissues, oxidative stress generation, thereby promoting liver fibrogenesis through hepatic stellate cell activation (98).JKB-121, a long-acting small molecule drug, mildly inhibits TLR4. *In vitro* experiments demonstrate its capacity to reduce or inhibit the release of inflammatory cytokines induced by LPS. It also diminishes the activity of hepatic stellate cells, suppresses their proliferation, and reduces collagen expression (104).During the second phase of clinical trials with JKB-121, the drug did not show statistically significant efficacy in improving fatty liver, liver enzymes, and metabolic biomarkers when compared to the placebo (105).

Considering the role of B cells in NAFLD progression, we postulate that B cell-targeted therapies may be robust modulators of disease progression. These approaches may encompass modulation of immunization, BCR signaling, disruption of B-cell survival and proliferation, as well as B-cell depletion. Consequently, targeting B-cell inflammatory mediators like TNFα holds promise as a potential therapeutic avenue (85). Alternatively, researchers could investigate the efficacy of monoclonal antibodies, antibiotics, or probiotic interventions in addressing polycytokine-producing CD4+ T cells as innovative options for managing NASH in patients (106).

Across multiple preclinical studies utilizing mouse models of diet-induced and genetic NAFLD/NASH, as well as isolated primary human cells, evidence indicates targeting components of both the innate and adaptive immune system can mitigate disease progression. Modulating signaling pathways and cells that promote inflammation, while restoring or enhancing regulatory immune cells and processes that resolve inflammation and clear apoptotic cells, appear to have therapeutic potential. Though mechanisms and specific targets differ, immunomodulatory approaches such as those targeting macrophages, T cells, B cells, and various inflammatory signaling molecules demonstrate efficacy in reducing steatosis, inflammation and early indications of anti-fibrotic effects. While additional research is warranted, particularly human clinical trials, current studies indicate that immunomodulation is a promising therapeutic avenue for NAFLD/NASH that may usefully complement other targets addressing metabolic dysfunction and injury processes not driven by immunopathology.

## 5 Conclusions

NAFLD is a common liver disease globally, which encompass a spectrum of conditions from basic steatosis or NAFL to NASH, NASH-linked fibrosis, cirrhosis, and HCC. Research on liver immunology has led to significant understanding of the contribution of immune cells, particularly macrophages, to the progression of NAFLD. This validates that immune dysregulation plays a crucial role in driving the progression of NAFLD. The pathogenesis of NAFLD entails various immune cell populations, and the orchestrated communication among them may contribute to the development of the hepatic inflammatory milieu observed during the progression of NAFLD disease. A growing body of evidence suggests that mitochondrial metabolism is differentially involved in the pathogenesis of NAFLD in a number of ways, such as cytokinesis, oxidative stress, autophagy, and mitochondrial quality control. It is important to note that impaired mitochondrial metabolism is widely associated with NAFLD by mediating dysregulation of lipid metabolic homeostasis. The deeper understanding of the molecular structure of mitochondrial plasticity is still in its infancy, but it may provide new doors for the development of future physiological compounds with potential for clinical NAFLD therapy (106, 107).

However, this more systematic pathogenesis of NAFLD immunomodulation has not been extensively explored. Therefore, future investigations should employ different NAFLD mice models to explore this topic. With the emergence and application of cutting-edge technologies like single-cell RNA sequencing (sc-RNA seq) and time-of-flight mass spectrometry flow cytometry, this knowledge gap is expected to be closed in the foreseeable future. This will offer a more profound understanding of the dynamic accumulation and activation of immune cells, potentially paving the way for innovative therapeutic strategies and anti-NAFLD medications. Ultimately, these efforts aim to alleviate the global burden of NAFLD.

## Author contributions

ZG: Conceptualization, Visualization, Writing – original draft, Writing – review & editing. QW: Resources, Visualization, Writing – review & editing. PX: Resources, Visualization, Writing – review & editing. JW: Conceptualization, Resources, Visualization, Writing – review & editing. WL: Conceptualization, Data curation, Supervision, Writing – review & editing.
